# A glimpse at tumor genome evolution

**DOI:** 10.1186/gb-2011-12-s1-i10

**Published:** 2011-09-19

**Authors:** Elaine R Mardis

**Affiliations:** 1 The Genome Institute at Washington University School of Medicine, St. Louis, MO 63108, USA

## 

Next-generation DNA sequencing has dramatically affected cancer genomics efforts in several important ways. Although whole genome sequencing remains an analytical challenge, such efforts are yielding data that elucidate the myriad ways in which a genome can be influenced by single point mutations, focused insertions or deletions, and large structural alterations. In addition to cataloguing somatic alterations, various correlation analyses are indicating the genes whose alterations most profoundly determine patient outcomes, patient responses to therapeutics and other important aspects of disease biology. We have recently begun exploring how the digital nature of next-generation sequencing reads allows important information about tumor cell genomic heterogeneity to be inferred, revealing the earliest mutations and how the composition of the tumor cell mass changes over time under the influence of stressors such as chemotherapy.

